# Ninjinyoeito Has a Protective Effect on the Auditory Nerve and Suppresses the Progression of Age-Related Hearing Loss in Mice

**DOI:** 10.3389/fnut.2020.528864

**Published:** 2020-10-09

**Authors:** Takanori Kawashima, Kenji Harai, Nina Fujita, Ryuji Takahashi

**Affiliations:** Kampo Research Laboratories, Kracie Pharma, Ltd., Tokyo, Japan

**Keywords:** age-related hearing loss, cochlear, Ninjinyoeito (NYT), auditory nerve, stria vascularis, auditory brainstem response

## Abstract

Currently, there are limited reports available regarding the treatment and prevention of progressive age-related hearing loss. This is because age-related hearing loss is not a critical disease with direct fatalities and has several well-established countermeasures such as hearing aids and cochlear implants. This study evaluated the efficacy of Ninjinyoeito (NYT) in the treatment of age-related hearing loss. C57BL/6J mice were divided into three groups: baseline group, untreated group, and NYT-treated group, with the latter receiving NYT treatment for 2 months. The mice were fed with NYT extract mixed with 4% mouse normal chow. Hearing loss was confirmed by a reduction in intact cell density of the auditory nerve from the age of 5–7 months. The suppression of hearing loss with aging and decrease in the intact cell density of the auditory nerve were significant in mice fed with NYT for 2 months. NYT has been reported to improve blood flow and enhance mitochondrial activity and may exert its protective effects on spiral neurons through these mechanisms. There was no decrease in the size of the stria vascularis from the age of 5–7 months in C57BL/6J mice. The present model failed to reveal the effect of NYT on atrophy of the stria vascularis of the cochlear duct. In conclusion, NYT appears to have a protective effect on the auditory nerve and suppress the progression of age-related hearing loss by reducing age-related auditory nerve degeneration.

## Introduction

The World Health Organization estimates that 466 million persons worldwide have disabling hearing loss (6.1% of the world's population); among them, 432 million (93%) are adults (242 million men and 190 million women), whereas 34 million (7%) are children; approximately one-third of persons older than 65 years have disabling hearing loss (https://www.who.int/deafness/estimates/en/). In Japan, more than 15 million people older than 65 years have age-related hearing loss (1). There has been limited research on the treatment and prevention of progressive age-related hearing loss because deafness is not perceived as a critical disease directly associated with mortality. Moreover, there are several countermeasures available (hearing aids and cochlear implants) for managing diagnosed cases of deafness.

However, deafness not only leads to a reduced communication ability but also causes other symptoms, such as isolation, depression, dementia, and other comorbidities associated with age-related deafness (2, 3). Moreover, the Japan Hearing Instrument Manufacturers Association has reported an extremely low proportion of hearing aid users for deafness in Japan. This could be attributed to the inability to purchase a hearing aid because of the high cost (24%). Even if they have a hearing aid, they do not use it because of factors, such as discomfort (46%), a feeling of unnecessity (25%), and shame (19%). In addition to hearing aid use and cochlear implant placement, controlling deafness progression with age is equally necessary for the management of age-related hearing loss. These pathological changes are reportedly caused by mitochondria-derived reactive oxygen species that reduce cochlear blood flow and cause abnormal accumulation of mitochondrial DNA (4). In Oriental medicine (*Shang Han Lun*), reduced cochlear blood flow in patients with age-related deafness is attributed to declined kidney function; moreover, the blood flow can be restored with kidney supplements, which are similarly considered to improve deafness and tinnitus. A previous study demonstrated the beneficial effects of Ninjinyoeito (NYT) on aging and enhancement of the general well-being of elderly patients (5). However, no study has analyzed the effect of NYT on age-related hearing loss. Therefore, this study aimed to evaluate the usefulness of NYT for the treatment of age-related hearing loss through improved cochlear blood flow in mice.

## Materials and Methods

### Plant Materials

**Figure 1:**
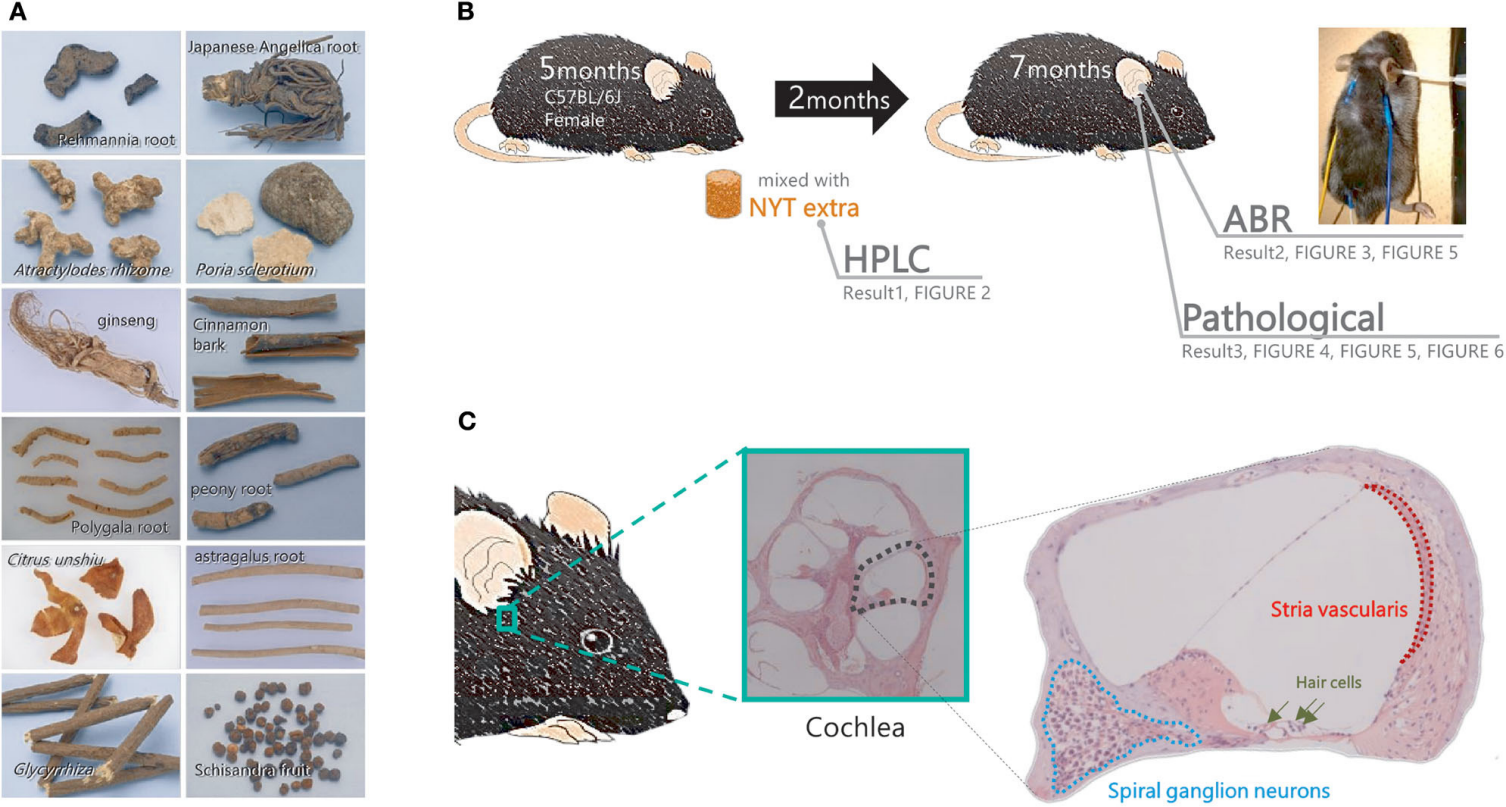
Flow of the experiment. **(A)** The component profile of the Ninjinyoeito (NYT) extract was evaluated using high-performance liquid chromatography. **(B,C)** Mice in the NYT-treated group were fed normal chow supplemented with 4% NYT, whereas those in the untreated group were fed normal chow for 2 months. After 2 months of treatment, the auditory brain response and pathological changes in the cochlea (spiral ganglion neurons and stria vascularis) were evaluated.

NYT is produced by Kracie Pharma (Toyama, Japan) as a powder of the dried *Rehmannia* root (4 g), Japanese angelica root (4 g), *Atractylodes* rhizome (4 g), *Poria sclerotium* (4 g), ginseng (3 g), cinnamon bark (2.5 g), polygala root (2 g), peony root (2 g), *Citrus unshiu* peel (2 g), astragalus root (1.5 g), *Glycyrrhiza* (1 g), and schisandra fruit (1 g) (Figure 1A). These plants are identified based on their external morphology and are authenticated based on marker compounds according to the Japanese Pharmacopeia as well as using our company's standards. The powder (lot no. E1712111A0) was mixed at 4% (w/w) with normal chow.

### High-Performance Liquid Chromatography

NYT extract (0.5 g) was mixed and shaken with 50% methanol (50 mL) followed by extraction through ultrasonication for 30 min. The supernatant was filtered using a membrane filter (0.22 μm) and subjected to three-dimensional high-performance liquid chromatography (HPLC) fingerprint analysis. The HPLC system comprised an LC-30AD pump, an SPD-M30A diode array detector (Shimadzu, Kyoto, Japan), a YMC-Triart C18 column (ϕ3.0 × 150 mm; YMC Co., Ltd., Kyoto, Japan), and 0.2% phosphoric acid in water/0.2% phosphoric acid in CH_3_CN (6:4) or 0.2% phosphoric acid in water/0.2% phosphoric acid in CH_3_CN (8:2) as solvent. In HPLC analyses, the flow rate was controlled by the LC-30AD at 0.5 mL/min. The eluent from the column was monitored, and the three-dimensional data were processed by the SPD-M30A diode array detector.

### Animals

Five-month-old female C57BL/6J mice were purchased from the Oriental Yeast Company, Ltd. (Shiga, Japan) and were acclimated for 1 week at 23 ± 3°C under a 12-h light/dark cycle (lights on from 08:00 to 20:00), with *ad libitum* access to chow and water. All efforts were made to minimize the suffering and the number of animals used. All mice were treated following the guidelines presented in the Standards for Human Care and Use of Laboratory Animals of Tohoku University and Guidelines for Proper Conduct of Animal Experiments by the Ministry of Education, Culture, Sports, Science, and Technology of Japan. All animal experiments were approved by the Ethics Committee for Animal Experiments of Tohoku University Graduate School of Medicine and the Experimental Animal Care Committee of Kracie Pharma. The approval number for the animal experiments was 2018MdA-189-1.

### Reagents and Mouse Diets

Ketamine was purchased from the Daiichi Sankyo Company Limited (Tokyo, Japan), and xylazine was purchased from the Fujifilm Wako Pure Chemical Corporation (Osaka, Japan). Mouse normal chow (MF) was purchased from CLEA Japan Inc. (Tokyo, Japan). Five-month-old female C57BL/6J mice were treated for 2 months with NYT powder mixed at 4% (w/w) with normal chow until 7 months of age (*n* = 10). The non-treated mice were fed MF.

### Model for Age-Related Hearing Loss

Five-month-old female C57BL/6J mice were administered NYT powder mixed at 4% (w/w) with normal chow. As controls, the non-treated mice received a regular diet of MF. After 2 months of NYT treatment, a hearing assessment was performed under anesthesia (7-month-old mice). The female C57BL/6J mice were classified into three groups. A hearing assessment was conducted in 10 mice at 5 months of age to establish a baseline. The control group of non-treated mice received a regular diet of MF for 2 months (*n* = 10) before the hearing assessment. The remaining 10 mice were administered NYT powder mixed at 4% (w/w) with normal chow from the age of 5–7 months. After 2 months of NYT treatment, a hearing assessment was performed under anesthesia. After undergoing the auditory brainstem response (ABR) assessment described below, the mice were sacrificed, and their cochlear tissues were collected for analysis (Figure 1B).

### Hearing Assessment

ABR hearing assessment was conducted as previously described (6). The mice were anesthetized by an intraperitoneal administration of ketamine (100 mg/kg body weight) and xylazine (20 mg/kg body weight). ABR was assessed in a soundproof room. For hearing threshold evaluation, three subdermal electrodes (ground, reference, and active electrodes) were placed 2–3 mm under the skin. The active electrode was subdermally inserted on the forehead (Figure 1B: yellow). The reference and ground electrodes were inserted below the pinna of the right ear (Figure 1B: blue) and back (Figure 1B: white). ABR recordings were obtained using a TDT System 3 auditory-evoked potential workstation and analyzed using the BioSigRP software (Tucker-Davis Technologies, Alachua, FL, USA). The ABR responses were evoked using bursts of pure tones at frequencies of 4, 8, 12, 16, and 32 kHz. Evoked responses were averaged across 1,000 sweeps. The responses were collected for stimulus levels in 5-dB steps from 100 to 10 dB SPL. The threshold shift was defined as the lowest sound intensity that could elicit at least one peak in the averaged ABR. In this experiment, we used wave V. Under blinded conditions, the ABR threshold was determined with a clear confirmation of wave V from the raw data. This was performed through double-checks by the person in charge and the blinded caregiver.

### Cochlear Tissue Analysis

After the ABR hearing assessment, cochlear tissues were collected from the mice (Figure 1C). After specimen collection, the perilymph was replaced with 4% paraformaldehyde, which was injected from the oval or round window with a syringe; subsequently, the cochlea was fixed. Thereafter, decalcification treatment using 10% ethylenediaminetetraacetic acid was performed, followed by stepwise dehydration with 70–100% ethanol. Subsequently, a paraffin block was prepared, and thin histological sections were obtained. Deparaffinization was performed with xylene, which was subsequently removed with ethanol. The slide was washed with water. The cells were stained with Meier hematoxylin for 5 min and washed with water for 30 s. The cells were soaked in a phosphate-buffered saline for 1 min and washed with water for 2 min. Eosin staining was performed for 1 min followed by washing of the cells with water and encapsulated. The intact cell density and area of the stria vascularis of the auditory nerve in the basal turn of the cochlea were measured.

### Statistical Analysis

All statistical analyses were performed using EZR (Saitama Medical Center, Jichi Medical University, Saitama, Japan), which is the graphical user interface for R-2.3-0 (The R Foundation for Statistical Computing, Vienna, Austria). All data are expressed as mean ± standard error of the mean.

Statistical comparisons were performed using one-way analysis of variance, followed by Tukey's test. Steel–Dwass test was used for data for which the *p*-value of the Bartlett test was <0.01 or for which the *p*-value of the Kolmogorov–Smirnov test (Shapiro–Wilk normality test) was <0.05.

Differences with *p* < 0.05 were considered statistically significant.

## Results

### High-Performance Liquid Chromatography of NYT Extract

**Figure 2:**
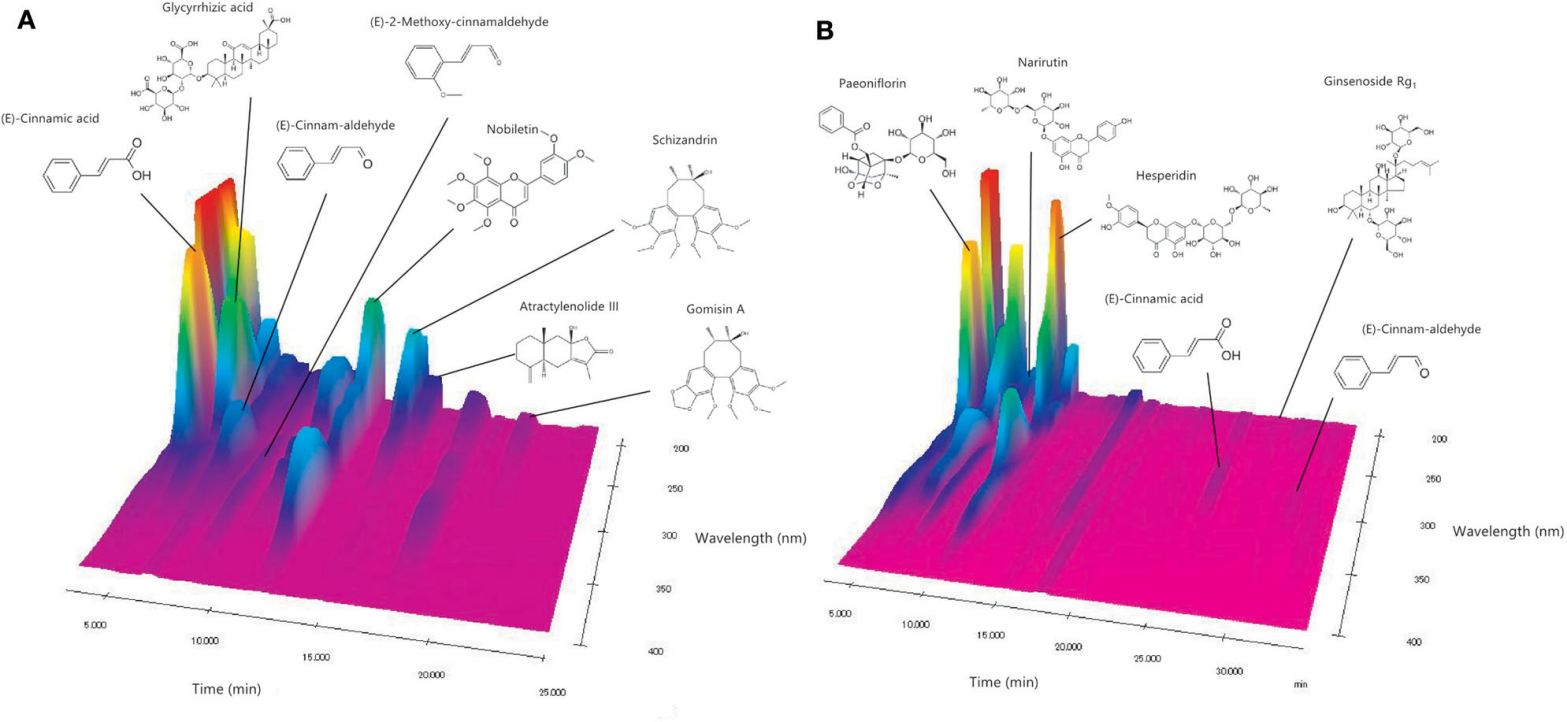
Analysis of NYT components. **(A,B)** The chemical compounds were identified in the chromatograms based on the comparison of their retention times and ultraviolet spectra (240–380 nm) with corresponding reference standards.

Figure 2 shows the chromatographic profile and composition of NYT. Chemical compounds were identified in the chromatographic profile according to their retention times and ultraviolet spectra, based on the corresponding reference standards. Specifically, (*E*)-cinnamic acid, glycyrrhizic acid, (*E*)-cinnam-aldehyde, (*E*)-2-methoxy-cinnamaldehyde, nobiletin, schizandrin, atractylenolide III, and gomisin A were identified when 0.2% phosphoric acid in water/0.2% phosphoric acid in CH_3_CN (6:4) was used as solvent (Figure 2A). Contrastingly, paeoniflorin, narirutin, hesperidin, (*E*)-cinnamic acid, ginsenoside Rg_1_, and (*E*)-cinnam-aldehyde were identified when 0.2% phosphoric acid in water/0.2% phosphoric acid in CH_3_CN (8:2) was used as solvent (Figure 2B).

### Hearing Assessment

**Figure 3:**
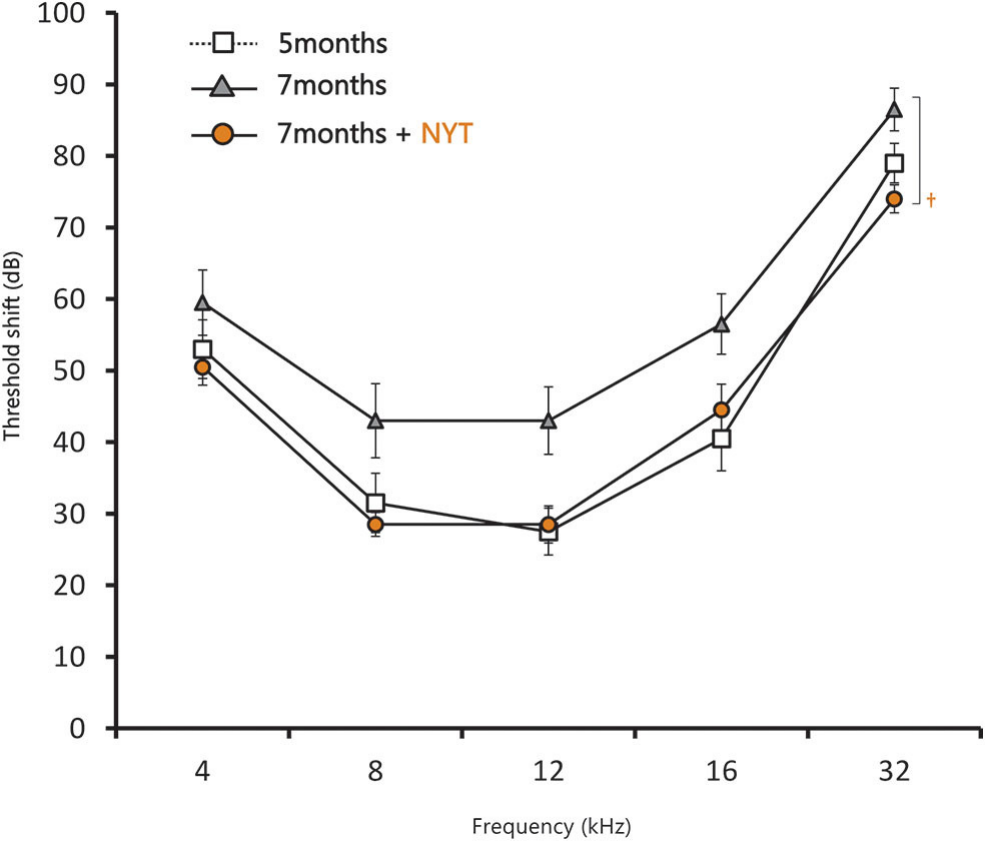
Results of the auditory brain response assessment after 2 months of NYT treatment. Data are expressed as mean ± standard error of the mean (*n* = 10). ^†^*p* < 0.05 vs. group of mice treated for 7 months (Steel–Dwass test).

In the ABR evaluation of female C57BL/6J mice after NYT administration, the threshold shift increased from 5 to 7 months of age, which confirmed decreased hearing. NYT was administered for 2 months from the age of 5 months; moreover, the hearing acuity was evaluated (Figure 3). Hearing acuity remained unchanged from the initiation of NYT administration at 5 months of age. Meanwhile, the hearing loss did not progress, and the hearing acuity at the age of 7 months was significantly higher in the NYT-treated mice than that in the untreated mice. Moreover, evaluation of the ABR results for each frequency revealed that hearing acuity was significantly higher in the NYT-treated mice than that in the untreated mice at 8, 12, and 32 kHz. The raw ABR data are presented in Supplementary Table 1.

### Analysis of Cochlear Tissue

**Figure 4:**
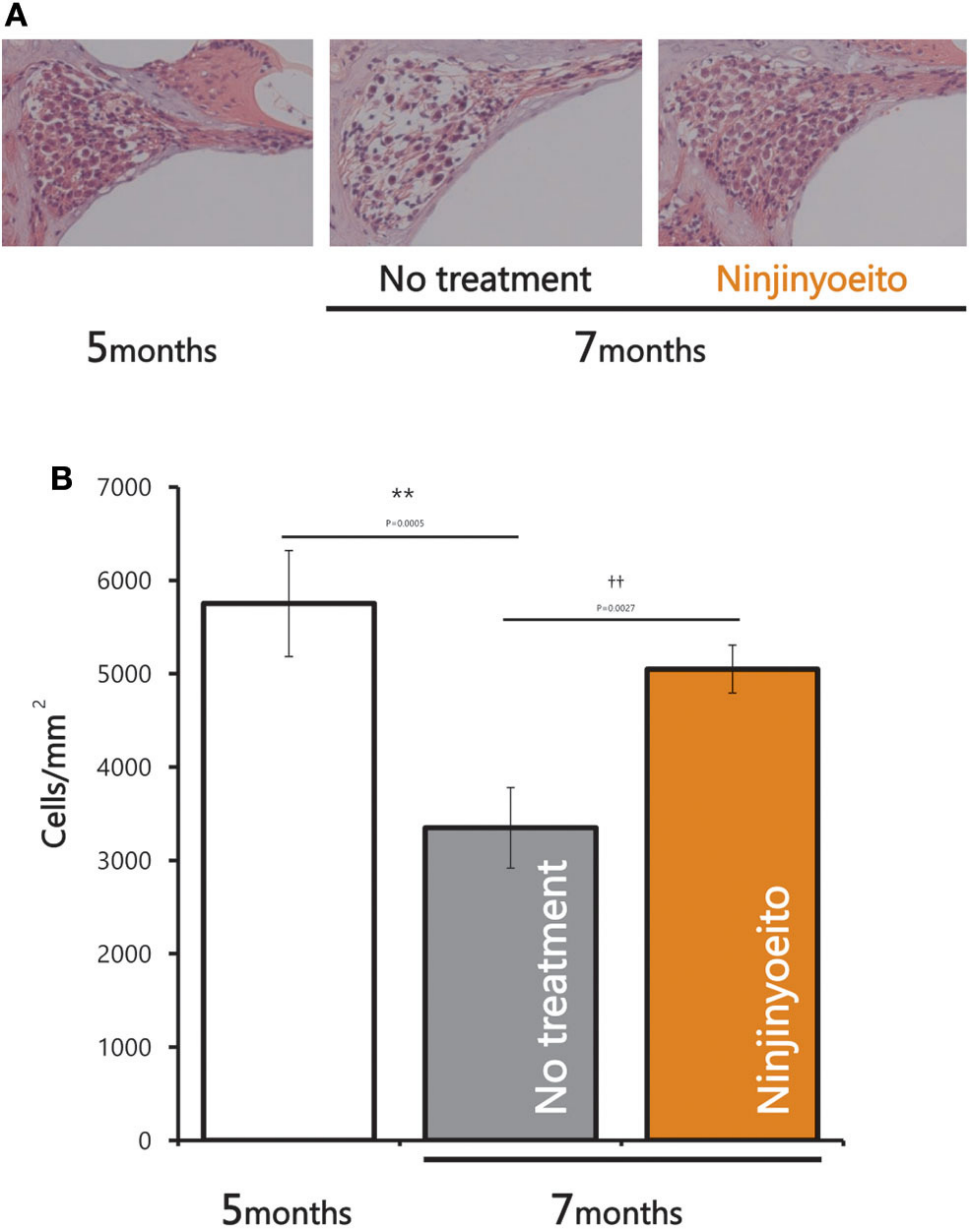
Pathological changes in spiral ganglion neurons after 2 months of NYT treatment. **(A)** Histological examination (hematoxylin and eosin stain) of specimens from each group. **(B)** Nerve cell viability (cells/mm). Data are represented as mean ± standard error of the mean (*n* = 7). **p < 0.01 vs. group of mice treated for 5 months; ^†^^†^*p* < 0.01 vs. group of mice treated for 7 months by Tukey's test.

**Figure 5:**
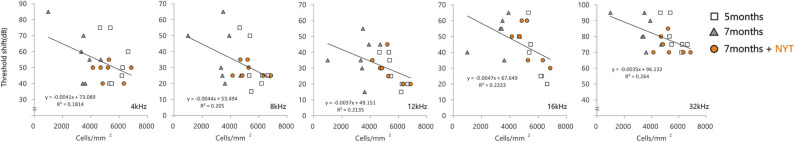
Correlation between hearing and nerve cell viability at each frequency (kHz).

**Figure 6:**
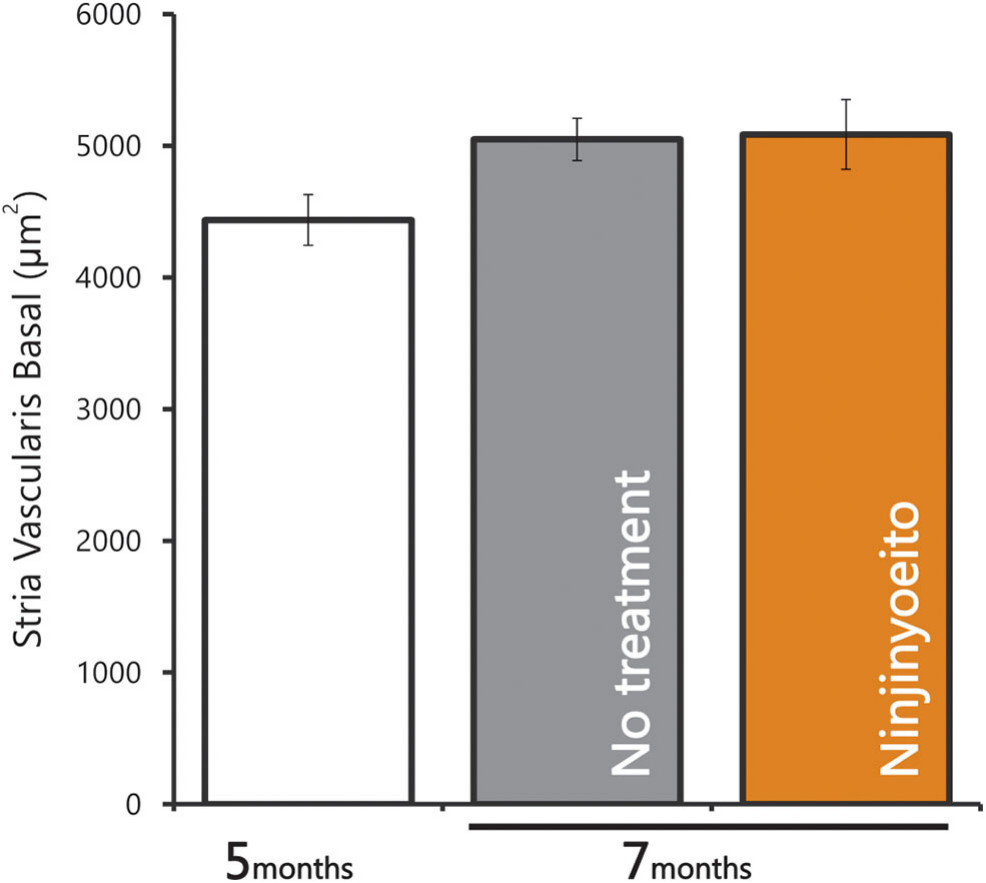
Area of the stria vascularis in the basal turn of cochlea. Data are represented as mean ± standard error of the mean (*n* = 7).

The intact cell density in the spiral ganglion was significantly reduced from 5 to 7 months of age. The intact cell density was significantly higher in the NYT-treated group than in the untreated group (Figures 4A,B). NYT seems to have a protective effect against atrophy and decreased auditory nerve excitation with aging. The spiral ganglion cell analyzed in this study was located in the basal turn of the cochlea and is associated with hearing acuity in the high-frequency auditory region. There was a marginally positive correlation between hearing acuity at a high frequency (16 kHz) and intact cell density of the spiral ganglion cells (cells/mm^2^) (*r* = 0.5; Figure 5). There was no decrease in the size of the stria vascularis from the age of 5–7 months (Figure 6).

## Discussion

This study reveals that NYT appears to suppress the progression of age-related hearing loss and protect the auditory nerve. Evaluation of the ABR results for each frequency revealed that the hearing acuity was significantly higher in the NYT-treated mice than that in the untreated mice at 32 kHz, which implies that NYT was effective in the high-frequency region characteristic of age-related hearing loss (Figure 3). The suppression of age-related hearing loss in NYT-treated mice could be attributed to a protective effect on the spiral ganglion. Age-related hearing loss is caused by the production of excess reactive oxygen species by the mitochondria due to age-related reductions in cochlear blood flow as well as the accumulation of abnormal mitochondrial DNA. NYT, which seemingly suppresses age-related hearing loss, is effective in improving blood flow. Additionally, it reportedly increases the mitochondrial activity and has a protective effect on the spiral ganglion cells. There was no decrease in the size of the stria vascularis from the age of 5–7 months (Figure 6). The present model failed to reveal the effect of NYT on atrophy of the stria vascularis of the cochlear duct. Various functional and structural changes that occur peripherally and centrally contribute to the development of age-related hearing loss, including degeneration of the stria vascularis, loss of hair cells and primary afferent neurons, and alterations in the central auditory pathways, including a reduction in the neurons of the cochlear nucleus and changes in neurotransmitter release (7). Age-related hearing loss supposedly results from aging, oxidative damage, mitochondrial impairment, and environmental factors (8, 9). Oxidative damage caused by reactive oxygen species has equally been postulated to play a causal role in age-related hearing loss (9–13). Since NYT is designed to improve fatigue, cold limbs, anorexia, night sweats, and anemia, it has been used for elderly people in traditional Oriental medicine. Specifically, NYT reportedly contributes to the improvement of peripheral circulation disorders (14, 15). Regarding ear disease, the Manabe group administered NYT to patients with acute, low-tone, sensorineural hearing loss. They hypothesized that NYT would improve the impaired blood supply to the stria vascularis and therefore be effective in patients with intractable acute, low-tone, sensorineural hearing loss (16). The C57BL/6J mice used in this study were reported to develop age-related hearing loss at 6 months (17) and have been widely used as an evaluation model for age-relating hearing loss (13, 18–23). In this study, we observed that NYT administration to 5-month-old C57BL/6J mice suppressed the progression of age-related hearing loss. The ABR results and the assessment of the association of intact cells suggest that NYT maintains hearing by protecting neurons in the cochlear nucleus that degenerate or die with age (Figure 5). It is speculated that NYT may have suppressed the progression of age-related hearing loss by improving the blood flow.

This study has several limitations. First, changes in cochlear blood flow could not be confirmed as being age-related. Currently, blood flow in the cochlea is evaluated based on a previous report by Kong's team (24). Second, the C57BL/6J mouse is an incomplete model for accurately reflecting the human age-related hearing loss. It is empirically known that men experience more rapid age-related hearing loss than women. However, in C57BL/6J mice, female mice develop age-related hearing loss much earlier and at a younger age than male mice. Another limitation is the short observation period, which was 2 months, as well as the administration of NYT before the increase in symptom severity. In the future, we plan to use CBA mice, which significantly correspond to the model of human age-related hearing loss (25). Third, in this experiment, it was technically impossible to evaluate changes in hair cells. Finally, this was only an observational study, and the molecular mechanism was not elucidated. HPLC results indicate that NYT contains cinnamaldehyde from cinnamon bark (Figure 2B). Cinnamaldehyde reportedly contributes to Nrf2 activation; however, this could not be verified in this study (26). In the future, we plan to evaluate whether NYT can cause Nrf2 activation and other underlying mechanisms.

Several components have been reported to be useful in preventing age-related hearing loss (11, 27). To our knowledge, food, or medicine that is useful for age-related diseases, such as fatigue, cold limbs, anorexia, night sweats, and anemia, has never been reported to suppress age-related hearing loss. NYT is covered by insurance. Since age-related hearing loss gradually progresses in all patients, NYT supplements for various geriatric problems, including progressive age-related hearing loss, can help enhance the general quality of life in the elderly population. In conclusion, NYT appears to have a protective effect on the auditory nerve and suppress the progression of age-related hearing loss by reducing age-related auditory nerve degeneration.

## Data Availability Statement

The datasets generated for this study are available on request to the corresponding author.

## Ethics Statement

This animal study was reviewed and approved by the Ethics Committee for Animal Experiments of Tohoku University Graduate School of Medicine the Experimental Animal Care Committee of Kracie Pharma.

## Author Contributions

TK designed the study and wrote the manuscript. TK and KH performed the experiments and analyzed the data. NF and RT revised the manuscript. All authors discussed the results and contributed to the final manuscript.

## Conflict of Interest

All authors are employees of Kracie Pharma (Toyama, Japan).

## References

[1] Uchida Y Sugiura S Nakashima T Ando F Shimokata H. [Estimates of the size of the hearing-impaired elderly population in Japan and 10-year incidence of hearing loss by age, based on data from the National Institute for Longevity Sciences-Longitudinal Study of Aging (NILS-LSA)]. Nihon Ronen Igakkai Zasshi. (2012) 49:222–7. 10.3143/geriatrics.49.222 23268872

[2] Gates GA Mills JH. Presbycusis. Lancet. (2005) 366:1111–20. 10.1016/S0140-6736(05)67423-5 16182900

[3] Lin FR Metter EJ O'Brien RJ Resnick SM Zonderman AB Ferrucci L. Hearing loss and incident dementia. Arch Neurol. (2011) 68:214–20. 10.1001/archneurol.2010.362 21320988 PMC3277836

[4] Lyu AR Kim TH Park SJ Shin SA Jeong SH Yu Y. Mitochondrial damage and necroptosis in aging cochlea. Int J Mol Sci. (2020) 21:2505. 10.3390/ijms21072505 32260310 PMC7177801

[5] Suzuki S Aihara F Shibahara M Sakai K. Safety and effectiveness of Ninjin'yoeito: a utilization study in elderly patients. Front Nutr. (2019) 6:14. 10.3389/fnut.2019.00014 30873411 PMC6401652

[6] Honkura Y Matsuo H Murakami S Sakiyama M Mizutari K Shiotani A. NRF2 Is a key target for prevention of noise-induced hearing loss by reducing oxidative damage of cochlea. Sci Rep. (2016) 6:19329. 10.1038/srep19329 26776972 PMC4726010

[7] Tavanai E Mohammadkhani G. Role of antioxidants in prevention of age-related hearing loss: a review of literature. Eur Arch Otorhinolaryngol. (2017) 274:1821–34. 10.1007/s00405-016-4378-6 27858145

[8] Kokotas H Petersen MB Willems PJ. Mitochondrial deafness. Clin Genet. (2007) 71:379–91. 10.1111/j.1399-0004.2007.00800.x 17489842

[9] Liu XZ Yan D. Ageing and hearing loss. J Pathol. (2007) 211:188–97. 10.1002/path.2102 17200945

[10] Seidman MD Khan MJ Bai U Shirwany N Quirk WS. Biologic activity of mitochondrial metabolites on aging and age-related hearing loss. Am J Otol. (2000) 21:161–7. 10.1016/s0196-0709(00)80003-4 10733178

[11] Darrat I Ahmad N Seidman K Seidman MD. Auditory research involving antioxidants. Curr Opin Otolaryngol Head Neck Surg. (2007) 15:358–63. 10.1097/MOO.0b013e3282efa641 17823554

[12] Yamasoba T Someya S Yamada C Weindruch R Prolla TA Tanokura M. Role of mitochondrial dysfunction and mitochondrial DNA mutations in age-related hearing loss. Hear Res. (2007) 226:185–93. 10.1016/j.heares.2006.06.004 16870370

[13] Someya S Xu J Kondo K Ding D Salvi RJ Yamasoba T. Age-related hearing loss in C57BL/6J mice is mediated by Bak-dependent mitochondrial apoptosis. Proc Natl Acad Sci USA. (2009) 106:19432–7. 10.1073/pnas.0908786106 19901338 PMC2780799

[14] Fujinami M Aotsuka S Okawa M Kinoshita M Sumiya M Thermographic assessment of Renshen-yangrong-tang (Ninjin-youei-tou) for the treatment of peripheral circulatory disturbance in patients with collagen disease [*In Japanese*]. Prog Med. (1980) 17:393–408.

[15] Takemiya T Clinical effects of ninjin-yoei-to in patients with peripheral circulation disturbance [*In Japanese*]. Jpn Pharmacol Ther. (1991) 19:409–16.

[16] Manabe Y Tokunaga T The effectiveness of Kampo medicines in the treatment of intractable acute, low-tone, sensorineural hearing loss. Otology Jpn. (2018) 28:139–43. 10.11289/otoljpn.28.139

[17] Kane KL Longo-Guess CM Gagnon LH Ding D Salvi RJ Johnson KR. Genetic background effects on age-related hearing loss associated with Cdh23 variants in mice. Hear Res. (2012) 283:80–8. 10.1016/j.heares.2011.11.007 22138310 PMC3277672

[18] Carlson S Willott JF. The behavioral salience of tones as indicated by prepulse inhibition of the startle response: relationship to hearing loss and central neural plasticity in C57BL/6J mice. Hear Res. (1996) 99:168–75. 10.1016/s0378-5955(96)00098-6 8970825

[19] Willott JF Bross LS. Morphological changes in the anteroventral cochlear nucleus that accompany sensorineural hearing loss in DBA/2J and C57BL/6J mice. Brain Res Dev Brain Res. (1996) 91:218–26. 10.1016/0165-3806(95)00188-3 8852372

[20] Johnson KR Erway LC Cook SA Willott JF Zheng QY A major gene affecting age-related hearing loss in C57BL/6J mice. Hear Res. (1997) 114:83–92. 10.1016/s0378-5955(97)00155-x 9447922

[21] Ison JR Allen PD O'Neill WE. Age-related hearing loss in C57BL/6J mice has both frequency-specific and non-frequency-specific components that produce a hyperacusis-like exaggeration of the acoustic startle reflex. J Assoc Res Otolaryngol. (2007) 8:539–50. 10.1007/s10162-007-0098-3 17952509 PMC2538342

[22] Vlajkovic SM Guo CX Telang R Wong ACY Paramananthasivam V Boison D. Adenosine kinase inhibition in the cochlea delays the onset of age-related hearing loss. Exp Gerontol. (2011) 46:905–14. 10.1016/j.exger.2011.08.001 21846498 PMC3200489

[23] Oike H Aoki-Yoshida A Kimoto-Nira H Yamagishi N Tomita S Sekiyama Y. Dietary intake of heat-killed *Lactococcus lactis* H61 delays age-related hearing loss in C57BL/6J mice. Sci Rep. (2016) 6:23556. 10.1038/srep23556 27000949 PMC4802309

[24] Kong TH Yu S Jung B Choi JS Seo YJ. Monitoring blood-flow in the mouse cochlea using an endoscopic laser speckle contrast imaging system. PLoS ONE. (2018) 13:e0191978. 10.1371/journal.pone.0191978 29489849 PMC5830291

[25] Sha S-H Kanicki A Dootz G Talaska AE Halsey K Dolan D. Age-related auditory pathology in the CBA/J mouse. Hear Res. (2008) 243:87–94. 10.1016/j.heares.2008.06.001 18573325 PMC2577824

[26] Uchi H Yasumatsu M Morino-Koga S Mitoma C Furue M. Inhibition of aryl hydrocarbon receptor signaling and induction of NRF2-mediated antioxidant activity by cinnamaldehyde in human keratinocytes. J Dermatol Sci. (2017) 85:36–43. 10.1016/j.jdermsci.2016.10.003 27720465

[27] Seidman MD. Effects of dietary restriction and antioxidants on presbyacusis. Laryngoscope. (2000) 110:727–38. 10.1097/00005537-200005000-00003 10807352

